# Exploring the Phytochemical Composition and Biological Potential of Balkan Endemic Species *Stachys scardica* Griseb

**DOI:** 10.3390/plants13010030

**Published:** 2023-12-21

**Authors:** Desislava I. Mantovska, Miroslava K. Zhiponova, Detelina Petrova, Kalina Alipieva, Georgi Bonchev, Irina Boycheva, Yana Evstatieva, Dilyana Nikolova, Ivanka Tsacheva, Svetlana Simova, Zhenya P. Yordanova

**Affiliations:** 1 Department of Plant Physiology, Faculty of Biology, Sofia University “St. Kliment Ohridski”, 8 Dragan Tsankov Blvd., 1164 Sofia, Bulgariazhiponova@biofac.uni-sofia.bg (M.K.Z.); d.petrova@biofac.uni-sofia.bg (D.P.); 2 Institute of Organic Chemistry with Centre of Phytochemistry, Bulgarian Academy of Sciences, bl. 9 Acad. Georgi Bonchev Str., 1113 Sofia, Bulgaria; kalina.alipieva@orgchm.bas.bg (K.A.); svetlana.simova@orgchm.bas.bg (S.S.); 3 Institute of Plant Physiology and Genetics, Bulgarian Academy of Sciences, Acad. Georgi Bonchev Str., Bl. 21, 1113 Sofia, Bulgaria; georgi.bonchev71@gmail.com (G.B.); irina_boycheva@bio21.bas.bg (I.B.); 4 Department of Biotechnology, Faculty of Biology, Sofia University “St. Kliment Ohridski”, 8 Dragan Tsankov Blvd., 1164 Sofia, Bulgaria; y.evstatieva@biofac.uni-sofia.bg (Y.E.); d.nikolova@biofac.uni-sofia.bg (D.N.); 5 Department of Biochemistry, Faculty of Biology, Sofia University “St. Kliment Ohridski”, 8 Dragan Tsankov Blvd., 1164 Sofia, Bulgaria; itsacheva@biofac.uni-sofia.bg

**Keywords:** ex situ conservation, in vitro culture, woundwort, growth regulators, antimicrobial activity, antioxidant activity, anti-inflammatory activity, phenylethanoid glycosides, iridoids

## Abstract

*Stachys scardica* Griseb. is a Balkan endemic species listed in The Red Data Book of Bulgaria with the conservation status “endangered”. Successful micropropagation was achieved on MS medium supplemented with 1.5 mg/L benzyladenine (BA), followed by a subsequent ex vitro adaptation in an experimental field resulting in 92% regenerated plants. Using nuclear magnetic resonance (NMR), phenylethanoid glycosides (verbascoside, leucosceptoside A), phenolic acids (chlorogenic acid), iridoids (allobetonicoside and 8-OAc-harpagide), and alkaloids (trigonelline) were identified, characteristic of plants belonging to the genus *Stachys*. High antioxidant and radical scavenging activities were observed in both in situ and ex vitro acclimated *S. scardica* plants, correlating with the reported high concentrations of total phenols and flavonoids in these variants. Ex vitro adapted plants also exhibited a well-defined anti-inflammatory potential, demonstrating high inhibitory activity against the complement system. Employing a disk diffusion method, a 100% inhibition effect was achieved compared to positive antibiotic controls against *Staphylococcus epidermidis* and *Propionibacterium acnes*, with moderate activity against *Bacillus cereus*. The induced in vitro and ex vitro model systems can enable the conservation of *S. scardica* in nature and offer future opportunities for the targeted biosynthesis of valuable secondary metabolites, with potential applications in the pharmaceutical and cosmetic industries.

## 1. Introduction

Since ancient times, plants have been providing humanity with effective remedies against various health disorders and inflammatory and infectious conditions. The development of modern science supports the effectiveness of medicinal plants and there is a rising trend in their application in primary health systems [1].

Being the largest and the outermost organ of the human body, the skin plays a main role in the interaction of the organism with the environment, and its main function is the protection of the body against harmful impacts like pathogens, pollutants, and unfavourable environmental conditions. All these factors disrupt skin homeostasis and induce oxidative stress and inflammation, which often results in the development of chronic skin diseases like psoriasis, atopic dermatitis, and eczema [2,3]. These are the most common inflammatory skin disorders in the human population and their conventional treatment includes the application of steroidal anti-inflammatory preparations that pose a risk of side effects [4].

Plant species are one of the most abundant sources of biologically active substances with anti-inflammatory, antibacterial, and antioxidant properties and thus have great potential for the development of natural products for the treatment of chronic skin diseases. The economic and pharmaceutical urge for such natural products makes the utilisation of medicinal plants more challenging as their natural populations could be negatively influenced. This requires the development of alternative strategies for the investigation, long-term conservation, and sustainable application of medicinal plants [5].

The genus *Stachys* or woundwort comprises more than 300 species and it is one of the largest genera from the Lamiaceae family. In Bulgaria, it is presented by 22 species, 5 of which are under the protection of Bulgarian biodiversity law. *Stachys* species are well known for their applications in traditional medicine for the treatment of different health disorders, infectious wounds, and skin inflammations. Different studies indicate that plants from the *Stachys* genus possess high antioxidant, anti-inflammatory, antimicrobial, and wound-healing properties due to their chemical profile [6,7]. The latest data on the phytochemical composition of various *Stachys* taxa show that the main chemical constituents of the genus are phenylethanoid glycosides, phenolic acids, flavonoids, iridoids, and, to a lesser extent, essential oils [8,9,10,11]. Numerous studies demonstrate that polar extracts from different *Stachys* species have great antioxidant capacity against free radicals like DPPH (2,2-Diphenyl-1-picrylhydrazyl) and FRAP (ferric-reducing antioxidant power), which correlates with the content of phenolics and flavonoids [12,13,14]. The anti-inflammatory potential of the genus has been studied through various in vitro and in vivo model systems. A polyphenol-enriched extract of *S. officinalis* was found to exhibit high inhibitory activities against COX-1 (cyclooxygenase 1) and LOX (lipoxygenase) enzymes [15]. The species *S. alpina*, *S. germanica*, and *S. recta* have been demonstrated to significantly inhibit inflammation in induced outflow in laboratory rats [16].

*Stachys scardica* Griseb. (The Plant List) or mountain woundwort is a Balkan endemic species included in The Red Data Book of Bulgaria with the conservational status “endangered”. It is distributed in Greece, Bosnia and Herzegovina, Serbia, and Montenegro. In Bulgaria, the populations of *S. scardica* are located mainly in the western mountains Osogovo and Konyavska [17]. There are no available data on the ex situ conservation of this species. It is reported that *S. scardica* contains phenylethanoid glycosides, mostly verbascoside [18].

The conservation status of mountain woundwort hinders its in-depth study, requiring the application of a biotechnological approach. The aim of our study was to perform ex situ conservation of *S. scardica* and a comparative study of the phytochemical composition and biological potential of methanolic extracts obtained from in vitro cultivated and ex vitro adapted plants. Utilising a biotechnological approach to induce in vitro and ex vitro cultures will contribute to the conservation of species while also providing an opportunity for the analysis and identification of substances responsible for their pharmacological potential. This, in turn, allows for an assessment of their practical applications in the pharmaceutical and cosmetic industries for the treatment of various skin disorders.

## 2. Results and Discussion

### 2.1. In Vitro Cultivation and Ex Vitro Acclimation of S. scardica

An increasing number of endemic plant species in Bulgaria characterised by pronounced medicinal properties face strong anthropogenic, biotic, and abiotic pressures that seriously threaten their abundance and diversity. The habitat of the endemic species *S. scardica* is concentrated in areas of Bulgaria where anthropogenic influence is intensifying. Despite measures taken by state institutions, such as including these species in protected areas as part of Natura 2000, their populations remain limited and scarce. The application of a biotechnological approach and the induction of *S. scardica* in in vitro cultures provide opportunities for its preservation and rapid propagation and the exploration of its pharmacological potential.

**Figure 1:**
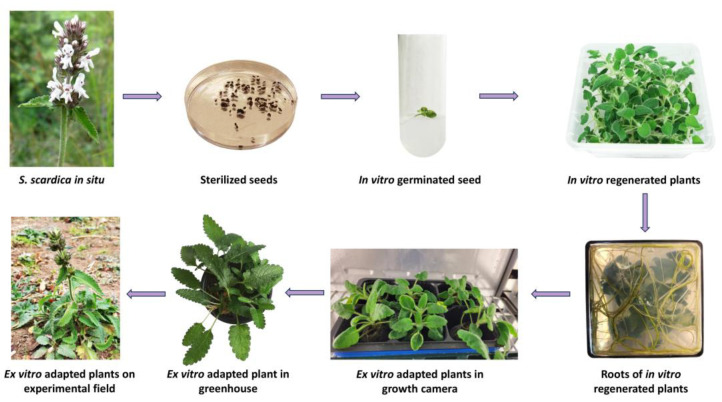
Induction of in vitro culture and ex vitro acclimation of the endemic species *S. scardica*.

Eighty sterilised seeds of *S. scardica* were evenly distributed on germination media: ½ MS (Murashige and Skoog) [19] and WA (water agar). After 30 days of incubation in the dark, an extremely low germination rate of 6.7% was observed only on WA. Further experiments involving different stratification periods and combinations of growth regulators were required to enhance the germination of *S. scardica* seeds [20,21,22]. In the subsequent stage, seedlings were transferred to a phytocultivation facility with controlled environmental conditions to prevent etiolation and support their normal development. After one month, the regenerated plants were micropropagated on MS medium without growth regulators. In vitro cultivated *S. scardica* plants exhibited well-developed aboveground and root systems. However, the plants produced a limited number of lateral shoots with elongated internodes, and each plant formed an average of 3.38 ± 1.11 new explants (Figure 1).

**Figure 2:**
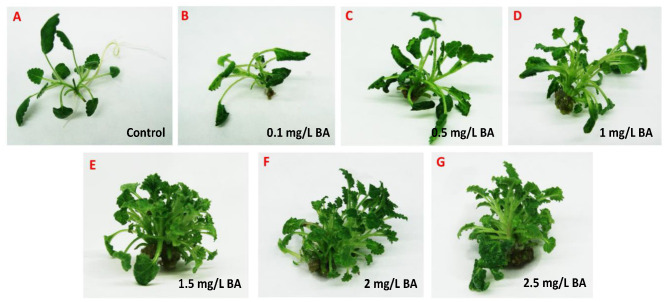
Influence of benzyladenine (BA) on the in vitro multiplication of *S. scardica*. (**A**) plant in vitro cultivated on MS control medium; (**B**–**G**) plants cultivated on MS medium supplemented with different concentration of BA (0.1–2.5 mg/L).

To enhance the growth index, the effect of the cytokinin benzyladenine (BA) on the in vitro propagation of *S. scardica* was investigated. Mono-nodal shoot segments from in vitro cultivated plants were inoculated on MS medium with various concentrations of BA (0.1, 0.5, 1.0, 1.5, 2.0, and 2.5 mg/L). After 28 days, all variants of MS medium supplemented with BA showed stimulation in the number of shoots formed from a single explant (Figure 2 and Table 1). The most effective medium for the in vitro propagation of *S. scardica* was with the addition of 1.5 mg/L of BA, where 80% of the plants formed shoots and produced 22.6 ± 2.47 new explants (Table 1). At higher concentrations of BA (above 1.5 mg/L), a significant increase in the growth index was not observed, and intensive callus formation was established. It is noteworthy that BA suppresses root formation, but when transferring the plants to MS medium without growth regulators, root formation was restored.

Benzyladenine has been successfully applied to increase the growth coefficient in other in vitro cultivated endemic species as well. In *Achillea thracica* and *Veronica caucasica*, 1 mg/L of BA stimulated growth under in vitro conditions, while in *Verbascum eriophorum*, 0.5 mg/L of BA stimulated the formation of 14.22 ± 0.2 shoots, without vitrification or callus formation being observed [23,24,25]. Panayotova et al. [26] and Mantovska et al. [11] reported the initiation of in vitro cultures of *S. maritima* and *S. thracica* where low seed germination was observed, but robust growth occurred on the medium without added growth regulators.

For ex vitro adaptation, 30 in vitro cultivated *S. scardica* plants with well-developed root systems were selected. The acclimatisation process was conducted through three successive stages—first within a growth chamber with controlled conditions followed by a greenhouse, ultimately resulting in a field trial. Initially, the in vitro cultivated plants were transferred to pots containing a sterile soil mixture and placed in a growth chamber under decreasing humidity conditions, changing weekly—90% > 80% > 70% > 60%. The survival rate of acclimated *S. scardica* plants appeared to be relatively high at 92%. Subsequently, the ex vitro regenerants were transitioned to greenhouse conditions and later planted in the experimental field. The plant survival percentage remained unchanged (Figure 1).

Micropropagation and ex situ conservation are successfully employed for rare plants with limited distribution and low seed germination rates [11,26]. The induction of in vitro and ex vitro collections of *S. scardica* has facilitated subsequent studies aimed at unravelling the pharmacological potential of this endemic species and comparing the metabolic profile of plants grown under different environmental conditions.

### 2.2. Taxonomic Position of S. scardica

**Figure 3:**
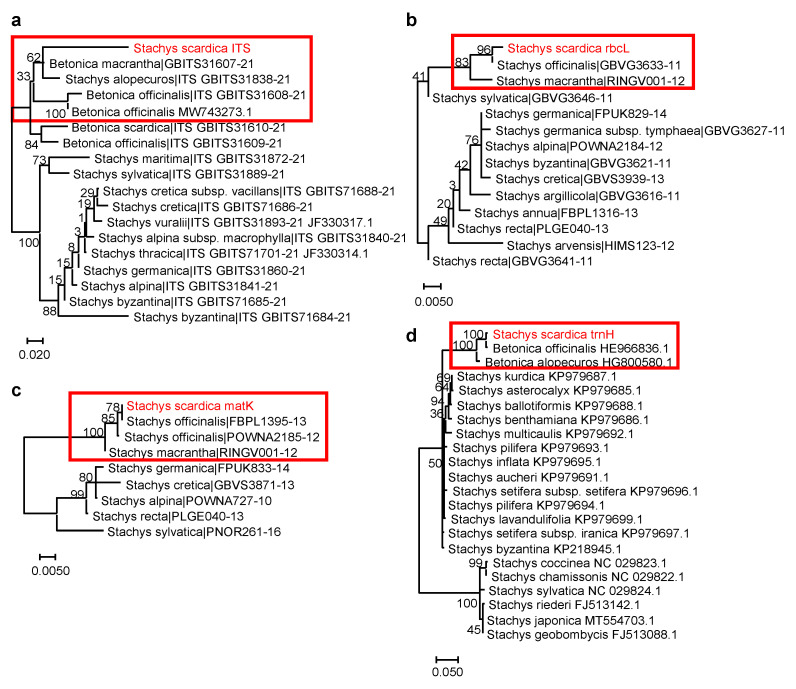
Phylogenetic position of *S. scardica* based on DNA barcoding markers. (**a**) ITS marker (*18* sequences from 89 total BOLD *Stachys* and *Betonica* sequences; DNA fragment length: *537 bp*); (**b**) rbcL marker (*14* sequences from 104 total BOLD *Stachys* sequences; DNA fragment length: *346 bp*); (**c**) matK marker (*9* sequences from 45 total BOLD *Stachys* sequences; DNA fragment length: *711 bp*); (**d**) trnH marker (*20* sequences from 53 total NCBI *Stachys* sequences; DNA fragment length: *330 bp*). The percentage of replicate trees in which the associated taxa clustered together in the bootstrap test (500 replicates) are shown next to the branches. The analysed *S. scardica* sequences are depicted in red and rectangular frames highlight the species phylogenetically related to *S. scardica*. Accessions from databases are shown with their respective numbers.

Due to the high number of species with both morphological similarities and differences, the genus *Stachys* has a complex taxonomy [27]. The whole genus is separated into two major subgenera—*Stachys* and *Betonica* L.—and each of them is further divided into sections—19 and 2, respectively [27,28]. The DNA barcoding approach has been developing rapidly and is a convenient tool for biodiversity studies, molecular phylogeny, and the taxonomy of medicinal plants [29,30,31]. Here, we applied this method to support taxonomic identification and define the phylogenetic position of *S. scardica*. The generated DNA barcode sequences were submitted to the BOLD database (https://www.boldsystems.org/index.php/Public_RecordView?processid=BUL003-23; accession number BUL003-23, accessed on 10 October 2023. We noticed a lack of other accessions in databases belonging to the species *S. scardica*. The performed sequence analyses allowed for the generation of *S. scardica* phylogenetic trees (Figure 3; Table 2).

Accordingly, *S. scardica* was assigned to the subgenus *Betonica* (L.) Bhattacharjee (=*Stachys* section Betonica Bentham). A close relation of *S. scardica* was observed with the “wood betony” *S. officinalis* (L.) Trevis. (or *B. officinalis*) [32], the “large-flowered”/“big” *S. macrantha* C. Koch. (or *B. macrantha*), and the “yellow betony” *S. alopecuros* (L.) Benth. (or *B. alopecuros*) [9,33]. Interestingly, the chloroplast markers (rbcL, matK, and trnH) showed 100% identity between *S. scardica* and *S. officinalis/B. officinalis* (Table 2). However, the application of the nuclear ITS marker managed to distinguish between the two species. Similarly, increased divergence between *S. scardica* and *S. macrantha/B. macrantha* was observed when using ITS compared to rbcL and matK. The more enriched ITS database also included *S. alopecuros* as a close species, as confirmed by trnH—again, ITS showed better phylogenetic resolution. The overall data highlighted the ITS marker as most appropriate for the taxonomic discrimination of *S. scardica*.

### 2.3. The Genetic Stability of In Vitro Cultivated and Ex Vitro Adapted S. scardica Plants

The evaluation of genetic stability is an important step during in vitro cultivation and subsequent ex vitro adaptation of medicinal plants due to the high possibility of somaclonal variations that result in morphological, cytological, biochemical, and genetic changes [34]. For monitoring genetic heterogeneity, different molecular methods are applied as they are independent of environmental factors and provide reliable and reproducible results. The SRAP approach developed by Li and Quiros [35] is an effective, simple, and adaptive marker system that can be used for various purposes. It has been successfully applied in the assessment of genetic diversity in natural populations of medicinal plant species from the Lamiaceae family like the *Thymus* species, *Lavandula angustifolia*, and *Origanum vulgare* [36,37,38]. SRAP analyses have been useful in the evaluation of genetic fidelity in in vitro cultivated plants like *Passiflora edulis* [39], and a recent report demonstrated the application of SRAP markers for confirming the genetic identity of in vitro and ex vitro adapted *S. thracica* plants [11]. In the current research, 16 combinations of SRAP primer pairs were used for the generation of fragments from *S. scardica* plant variants. Overall, the presence of 587 different alleles was established, with no variations between the SRAP profiles of in vitro cultivated and ex vitro adapted plants, which is an indication of preserved genetic stability in the process of micropropagation (Table S1).

### 2.4. NMR Fingerprinting during S. scardica Ex Situ Conservation

To monitor the alterations in metabolite profiles during the ex situ conservation of *S. scardica*, a comparative metabolite analysis of in situ, in vitro cultivated, and ex vitro adapted plants was conducted using NMR (nuclear magnetic resonance). A total of 17 compounds were identified, spanning both primary and secondary metabolites of *S. scardica*, including amino acids, sugars, organic acids, phenolic compounds, iridoids, and alkaloids (Table 3). In all three variants, an accumulation of sugars such as sucrose and α- and β-glucose was observed. Among the amino acids, alanine and glutamine were identified, with the latter found solely in the in vitro cultivated plants. The presence of glutamine in in vitro cultivated plants was likely due to the high concentrations of NH_4_NO_3_ (21 mM) and KNO_3_ (19 mM) as nitrogen sources in the in vitro cultivation medium. Previous studies indicated that increased nitrate has a beneficial effect, stimulating the synthesis of amino acids and proteins, while excess ammonium ions can be toxic and promote amide formation [40]. Recent research suggests that the non-protein amino acid β-alanine may accumulate in cells as a generic stress response molecule, participating in plant defence against temperature shock, hypoxia, drought, heavy metals, and biotic stress [41].

In the examined variants, several low molecular weight organic acids were also identified, including acetic, lactic, succinic, formic, and malic acids. Acetic, succinic, and formic acids were found in both in situ and ex vitro *S. scardica*, while lactic, formic, and malic acids were identified in in vitro cultivated plants (Table 3). It has been reported that under stress and adaptation to changing environmental conditions, plants respond with an increased biosynthesis of organic acids and their exudation into the soil through the roots. Plants are observed to secrete organic acids to mobilise phosphorus uptake in deficient soils [42]. Since these molecules are chemically charged, they can balance ion excess in cells and thus play a critical role in regulating cellular pH and osmotic potential [43,44]. Their charge makes them excellent metal chelators in a wide variety of environments. Succinic acid has been found to participate in binding metal cations and releasing phosphorus from bound complexes [45] and may also influence primary root growth during phosphorus deficiency [46]. The accumulation of acetic acid has been observed in plants subjected to drought and oxalate during biotic stress [47,48].

In the extracts of *S. scardica*, secondary metabolites that are typical representatives of the genus *Stachys* were identified—phenylethanoid glycosides, iridoids, phenolic acids, and alkaloids [8]. The phenylethanoid glycoside verbascoside and chlorogenic acid were detected in all three variants, while leucoseptoside A and trigonelline were found in ex vitro and in situ samples. Signals for allobetonicoside and 8-OAc-harpagide were observed only in the spectra of in vitro cultivated plants. The identification of phenylethanoid glycosides and iridoids was confirmed by comparing the NMR spectra with spectra of authentic samples isolated from the species *Sideritis scardica* and *Lamiastrum galeobdolon*.

One of the primary physiological roles of plant secondary metabolites is to mediate their interaction with the environment, and their content changes under the influence of various factors such as high and low temperatures, light exposure, pathogens, herbivores, etc. Changes in the metabolic composition of *S. scardica* cultivated under different environmental conditions indicate the significance of various compounds in the adaptation of individual species, both from in situ to in vitro and from in vitro to ex vitro conditions. The obtained results suggest that the content of verbascoside remains unchanged depending on the cultivation conditions, implying that other compounds are responsible for plant adaptation. However, a different trend was observed for iridoids. In *S. scardica*, allobetonicoside and 8-OAc-harpagide were identified only in in vitro cultivated plants. It has been observed that in vitro cultivated *Scrophularia takesimensis* exposed to blue LED light accumulated more harpagoside compared to those grown under white fluorescent light and red LED light [49]. It had been presumed that harpagoside likely protects plants from UVB rays. It has also been demonstrated that harpagide is mainly found in the stems of *Scrophularia* spp., while its derivative, harpagoside, accumulates in the leaves at higher temperatures. According to [50], harpagide is most likely transported through the phloem to the leaves, where its derivatives, harpagoside and acetylharpagide, are synthesised.

Verbascoside is one of the most characteristic secondary metabolites for the genus *Stachys*. Verbascoside and leucoseptoside A have also been identified in *S. officinalis*, *S. recta*, *S. affinis*, *S. alpina* subsp. *dinarica*, *S. anisochila*, *S. beckeana*, *S. byzantine*, *S. plumose*, *S. iva*, *S. candida*, *S. schtschegleevii*, *S. thracica*, etc. [8,11,51]. Both compounds are characterised by a wide range of biological activities including antioxidant, anti-inflammatory, hepatoprotective, anti-diabetic, and others [52].

Chlorogenic acid emerged prominently in the NMR spectra of *S. scardica* extracts, highlighting its significance. This biologically active phenolic acid is a distinguishing feature of the Asteraceae and Lamiaceae plant families [53]. Renowned for its diverse therapeutic attributes, chlorogenic acid demonstrates antioxidative, antibacterial, hepatoprotective, cardioprotective, anti-inflammatory, antipyretic, neuroprotective, and antiviral properties. Studies have indicated its potential to influence lipid metabolism and glucose levels, offering promise for addressing both genetic and acquired metabolic disorders [53].

Harpagide is considered a taxonomic marker for the genus *Stachys*. In recent years, this compound has attracted interest from various researchers due to its potential application as an anti-inflammatory agent. It has been found that after hydrolysis, the obtained compound not only inhibits the expression of the COX-2 enzyme but also reduces the prostaglandins produced by it, acting as a competitive inhibitor. In addition to its anti-inflammatory activity, harpagide has demonstrated antioxidant, neuroprotective, anti-tumour, spasmolytic, and antimicrobial activities [54]. Alobetonicoside is encountered less frequently and has been identified only in specific representatives of the genus *Stachys*, such as *S. macrantha*, *S. glutinosa*, and *S. officinalis* [55].

### 2.5. Comparative Determination of Total Phenols and Flavonoids in In Situ, In Vitro Cultivated, and Ex Vitro Adapted Plants

**Figure 4:**
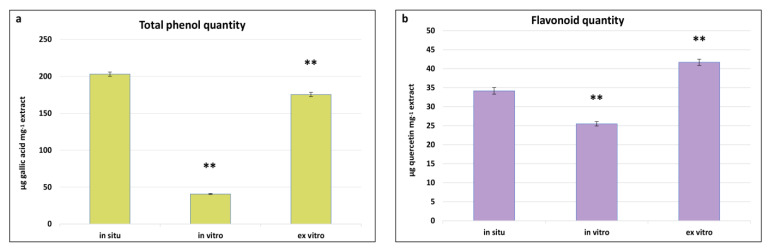
Total phenolic (**a**) and flavonoid (**b**) quantities in in situ, in vitro cultivated, and ex vitro adapted *S. scardica* plants. Mean values ± SD are shown. Significant changes are indicated with asterisk ** (*p* ≤ 0.001).

Phenolics represent one of the chemically diverse groups of secondary metabolites in plants, characterised by a variety of pharmacological properties such as antioxidant, anti-inflammatory, antimicrobial, anti-tumour, and cardioprotective activities [56]. As integral components of secondary metabolism, their biosynthesis and quantity are significantly influenced by environmental conditions. Determining the total content of phenols and flavonoids in in situ, in vitro cultivated, and ex vitro adapted plants of *S. scardica* provides insights into the changes in the levels of these metabolites in plants with the same genotype but cultivated under different conditions. The highest total phenol content was observed in in situ *S. scardica* plants, followed by ex vitro adapted plants—203.14 ± 2.7 and 175.5 ± 2.7 μg GA/mg extract, respectively (Figure 4). Conversely, the highest flavonoid content was established in ex vitro adapted plants, followed by in situ plants—41.6 ± 0.8 and 34.6 ± 0.8 μg Q/mg extract, respectively. In in vitro cultivated plants, the phenolic and flavonoid contents were considerably lower, likely attributable to the aseptic culture conditions.

Similar results were observed in other in vitro cultivated *Stachys* species—*S. thracica* and *S. bulgarica* [11,57]. The inhibition of secondary metabolite biosynthesis was also noted during the in vitro cultivation of *Nepeta nuda* [31], suggesting that environmental factors may diminish the biosynthetic capacity.

### 2.6. The Antioxidant and Radical Scavenging Activities of In Situ, In Vitro Cultivated, and Ex Vitro Adapted S. scardica Plants

Due to their structural features, phenolics are considered major antioxidant molecules. Their protective effect arises from the ability to donate hydrogen and thus “scavenge” free radicals. Numerous studies demonstrate that excessive oxidative damage significantly contributes to the development of skin disorders such as psoriasis, atopic dermatitis, and skin cancer [58,59,60,61]. There is substantial evidence that phenolic compounds like resveratrol, curcumin, and rosmarinic acid are effective in the treatment of skin diseases, and there is great interest in discovering novel compounds with antioxidant activities [62].

**Figure 5:**
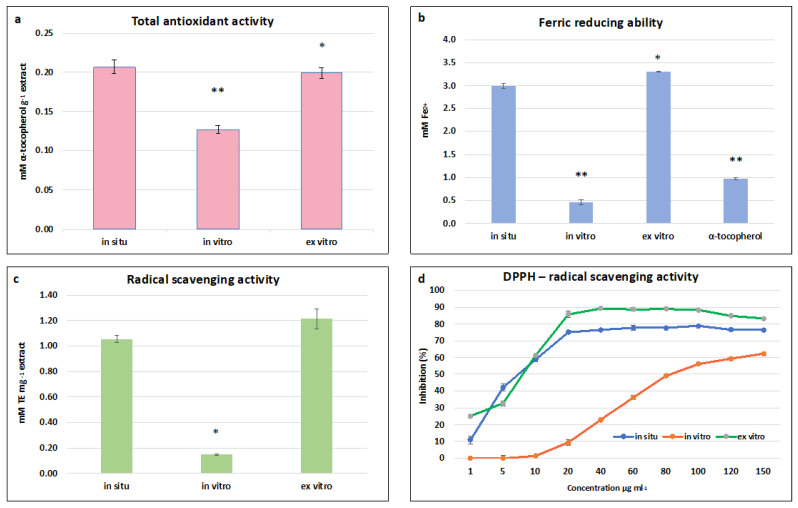
Antioxidant and radical scavenging potential of in situ, in vitro cultivated, and ex vitro adapted *S. scardica* plants. (**a**) Total antioxidant activity. (**b**) Ferric-reducing ability. (**c**) ABTS radical scavenging activity. (**d**) DPPH radical scavenging activity. Mean values ± SD are shown. Significant changes compared to in situ plants are indicated with asterisks ** (*p* ≤ 0.001), * (*p* ≤ 0.05).

The highest total antioxidant activity (TAA) was established in in situ and ex vitro adapted *S. scardica* plants—0.197 ± 0.006 mM·g^−1^ and 0.200 ± 0.007 mM α-tocopherol·g^−1^, respectively (Figure 5a). A two-fold decrease was observed in in vitro cultivated plants, which correlated with the total phenolic and flavonoid contents. A similar dependence was observed in the FRAP assay, where the highest ferric-reducing ability of ex vitro adapted plants was equal to that of in situ wild plants—3.3 ± 0.01 mM Fe ^2+^ and 3.0 ± 0.05 mM Fe ^2+^, respectively (Figure 5b). The activity of both variants was two times higher than the standard used—α-tocopherol. The highest ABTS radical scavenging activity was observed in ex vitro adapted plants, followed by in situ wild plants—1.21 ± 0.080 mg TE·g and 1.056 ± 0.029 mg TE·g, respectively (Figure 5c). A general trend was found that the radical scavenging capacity measured by FRAP and ABTS assays of in vitro cultivated plants was five times lower than the activity of in situ wild and ex vitro adapted *S. scardica*.

In the DPPH assay, all three extracts managed to scavenge the free radical in a concentration-dependent manner. The maximum inhibition of the radical was 90% and 77% at a concentration of 60 μg/mL for ex vitro adapted and in situ wild plants, respectively, and 62% at a concentration of 150 μg/mL for in vitro cultivated plants (Figure 5d). The lowest concentrations at which 50% inhibition of the DPPH radical was observed were 8.1 μg/mL and 8.5 μg/mL for ex vitro and in situ wild plants, respectively, and 81 μg.ml for in vitro cultivated plants.

The high antioxidant potential of the in situ wild and ex vitro adapted *S. scardica* plants is likely attributable to the key identified compounds in these extracts—verbascoside and chlorogenic acid (Table 3). It has been reported that phenylethanoid glycosides and phenolic acids are potent antioxidants capable of neutralising free oxygen forms directly or breaking the chain of peroxide radicals [13,51]. It has been established that the main compounds responsible for the high radical scavenging capacity of methanolic extracts from *S. officinalis* are verbascoside and chlorogenic acid, constituting approximately 69% of the total antioxidant activity of extracts [13].

Consistent with our findings, Vundac et al. [63] also suggested that the elevated antioxidant activity of the species *S. recta*, *S. salvifolia*, *S. officinalis*, *S. alpina*, *S. palustris*, and *S. sylvatica* is attributable to the presence of chlorogenic acid. Similarly, Sliumpate et al. [13] demonstrated that verbascoside and chlorogenic acid play a significant role in the radical scavenging activity of methanolic extracts from *S. officinalis*. Furthermore, various studies suggest that different crude plant extracts exhibit high antioxidant activity and radical scavenging potential primarily due to the presence of verbascoside and chlorogenic acid [64,65].

### 2.7. The Anti-Inflammatory Activity of S. scardica

The anti-inflammatory activity of the methanolic extracts from *S. scardica* plants was evaluated by a hemolytic assay that examined the effect of the extracts on the complement system via the classical pathway (CP). The complement system has an important role in the innate immune response in cases of bacterial infections or other stimuli. The activation of the complement system leads to an inflammatory response through the production of proinflammatory molecules [66]. However, if poorly regulated, continuous activation may occur, which leads to prolonged inflammation. Therefore, the inhibition of the complement system leads to an anti-inflammatory response. Different studies indicate that there is a relationship between the activation of the complement system and skin diseases like psoriasis, acne vulgaris, etc. [67,68].

**Figure 6:**
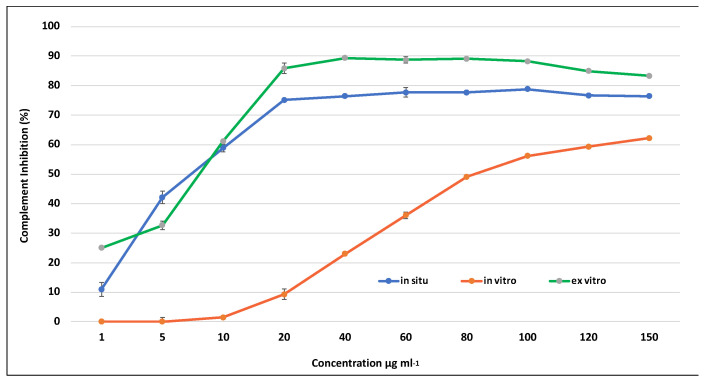
Inhibition of hemolysis by extracts of in situ, in vitro cultivated, and ex vitro adapted *S. scardica* plants. Mean values ± SD are shown.

All tested extracts effectively inhibited the complement system in a dose-dependent manner. Both the extracts derived from in situ wild and ex vitro adapted *S. scardica* plants exhibited comparable activity against the complement system, achieving a maximum inhibition of up to 96% at a concentration of 2000 μg/mL (Figure 6). In contrast, the activity of extracts from in vitro cultivated plants at the same concentration was nearly two-fold lower, reaching 57%, aligning with the observed variance in secondary metabolite content. The lowest observed concentration at which 50% inhibition of hemolysis (IC_50_) occurred was in ex vitro adapted plants at 227 μg/mL, followed by in situ wild plants at 840 μg/mL, and the highest IC_50_ was recorded for in vitro cultivated plants at 1785 μg/mL.

The extracts derived from in vitro cultivated plants demonstrated notably reduced anti-inflammatory activity. This suggests that the aseptic environment and cultivation conditions had a substantial impact on decreasing the concentration of phenolic compounds and, consequently, diminished the associated anti-inflammatory activity in in vitro cultivated *S. scardica* plants. Conversely, ex vitro conditions facilitated the restoration of biosynthetic capacity, resulting in an elevation of the biological activity in the plants.

In recent years, there has been growing interest in the research and development of drugs targeting the complement system. Various extracts from *Euphorbia umbellata* exhibit the ability to inhibit the complement system by up to 33%. Additionally, flavonoid-rich extracts from *Ligustrum vulgare* and *Phillyrea latifolia* demonstrate significant inhibitory activities [69]. The anti-inflammatory activities of different *Stachys* species have been extensively investigated using in vitro and in vivo models. A polyphenol-rich extract from *S. officinalis* exhibited strong inhibitory activities against the COX-1 and LOX enzymes [15]. Furthermore, it has been reported that flavonoid-rich extracts from *S. inflata* and *S. mialhesi* displayed high anti-inflammatory potential in a carrageenan-induced rat paw edema model [70,71].

### 2.8. The Anti-Microbial Activity of S. scardica

Due to the excessive and often improper use of antibiotic drugs, there has been an observed resistance in most bacterial strains causing infections in humans in recent years. Antibiotic resistance is a global issue, and its resolution necessitates the increasingly intensified development of alternative antibiotic products [72]. The use of plants from the *Stachys* genus for treating various infectious diseases, including wound healing, indicates that the synthesised metabolites from these species indeed exhibit antimicrobial activities. In recent years, the scientific literature has been enriched with evidence supporting this [7,14,22,73].

Methanolic extracts obtained from in vitro cultivated and ex vitro adapted *S. scardica* plants were tested against the skin pathogens *Staphylococcus epidermidis*, *Propionibacterium acnes* (an isolate), *Bacillus cereus*, *Escherichia coli*, *Candida albicans*, and *Malassezia furfur* by applying the disc diffusion method. The methanolic extracts from ex vitro acclimated *S. scardica* showed strong inhibition against *S. epidermidis* and *P. acnes*, with a 100% inhibition effect compared to the positive controls gentamicin and clindamycin, respectively (Table 4). Moderate activity against *B. cereus* was established. It is noteworthy that the extracts isolated from the ex vitro adapted plants had nearly three times higher inhibitory activities compared to those from the in vitro cultivated plants. None of the studied extracts had antimicrobial activities against the tested *E. coli*, *C. albicans*, or *M. furfur*.

The most likely reason for the observed antibacterial activity of *S. scardica* was the presence of verbascoside in the isolated extracts. It has been reported that verbascoside exhibits activity against *P. mirabilis* and *S. aureus* at concentrations of 64 μg/mL and 128 μg/mL, respectively [74]. These results were confirmed by Souza et al. [75], who found the activity of this secondary metabolite against *S. aureus* and *S. epidermitis* at concentrations of 63 μg/mL and 32 μg/mL, respectively. Similar results have been reported by Agampodi et al. [76], indicating that verbascoside has the potential to inhibit the growth of *S. aureus*, *S. epidermitis*, *P. aeruginosa*, *P. mirabilis*, and *A. baumanii* at concentrations of 9.77 μg/mL, 9.77 μg/mL, 1250 μg/mL, 312.5 μg/mL, and 1250 μg/mL, respectively.

## 3. Materials and Methods

### 3.1. Chemicals

Murashige and Skoog medium, plant agar, and sucrose used for in vitro cultivation were purchased from Duchefa Biochemie (Haarlem, The Netherlands). The kit for DNA extraction was purchased from Qiagen (Hilden, Germany). The organic solvents used for extraction, the reagents, and the standards used for the determination of total phenols and flavonoids, as well as the DPPH, ABTS, and FRAP free radicals, were purchased from Sigma-Aldrich (Madrid, Spain). CD_3_OD and D_2_O came from Deutero GmbH (Kastellaun, Germany). The sensitised red sheep erythrocytes, rabbit hemolysin, and guinea pig serum were purchased from BulBio (Sofia, Bulgaria).

### 3.2. Plant Material—In Vitro Cultivation and Ex Vitro Adaptation

The plant material from in situ grown *S. scardica* plants was collected from their natural habitat—near Kyustendil province in the period of blooming in July, with seeds collected in September, with the permission of the Bulgarian Ministry of Environment and Water. A voucher specimen SO108162 was deposited at the Herbarium of Sofia University “St. Kliment Ohridski”. The in vitro shoot culture was induced by the sterilisation of dried ripe seeds with 70% ethanol and incubated on germination medium [11]. After germination, the seedlings were cultured on full-strength MS medium [19] and cultivated in a phytochamber under controlled environmental conditions (80 μmol m**^−^**^2^ s**^−^**^1^ photosynthetic active radiation, cool white fluorescent TL-D36W/54-765 1SL/25 Philips, photoperiod 16 h light/8 h dark, 25 °C, 70–80% moderate humidity). The plants were micropropagated twice over a period of 30 days before performing further experiments.

In vitro cultivated plants featuring regenerated shoots measuring 3–4 cm in length with 2–3 internodes and a well-established root system underwent ex vitro adaptation. The regenerated plants were transplanted into plastic pots filled with a sterile soil substrate mixture (peat:coconut fibres:sand = 2:1:1). Acclimation was conducted over one month in a phytotron chamber (POL-EKO APARATURA SP.J.A. Polok—Kowalska KK 350 STD 1400 W) under conditions of 16/8 h light/dark, 100 µmol m^–2^ s^–1^ PPFD, and a temperature of 22 ± 2 °C. Relative humidity was gradually reduced from 90% to 60% each week. Following 30 days of acclimation, the adapted plants were moved to a greenhouse for an additional month before being transferred to regular garden soil at the experimental field of Sofia University “St. Kliment Ohridski”. After a year of acclimation to field conditions, newly formed, fully expanded leaves from the 2nd or 3rd nodes of the stem of ex vitro plants during the blooming period were harvested for subsequent analysis and NMR metabolic profiling.

### 3.3. DNA Barcoding Analysis

This procedure was performed according to Petrova et al. [31]. Genomic DNA was extracted from *S. scardica* in vitro plants using a DNeasy Plant Mini kit (Qiagen, Hilden, Germany) according to the manufacturer’s instructions. The taxonomic identification of *S. scardica* samples was performed through DNA barcoding based on the sequences of four gene regions: nuclear ribosomal internal transcribed spacer (ITS), ribulose-1,5-bisphosphate carboxylase/oxygenase large subunit (rbcL) gene, maturase K (matK) gene, and trnH-psbA intergenic spacer. The primer sequences (synthesised by Microsynth) and PCR conditions that varied among primers are shown in Table S2. PCR amplification was performed in 20 μL reaction mixtures containing approximately 30 ng of genomic DNA, 1× PCR buffer, MgCl_2_ (2.0 mM for ITS, 2 mM for matK, and 2.5 mM for rbcL and trnH-psbA), 0.2 mM of each dNTP, 0.2 μM of each primer, and 1.0 U Taq DNA Polymerase (Solis BioDyne, Tartu, Estonia). Amplicon products for all four gene regions were sequenced in both directions by Microsynth (Göttingen, Germany) with the same primers used for PCR amplification. Candidate DNA barcode sequences for each barcode region were edited and aligned in the software package Molecular Evolutionary Genetics Analysis (MEGA) ver. MEGA X Kumar et al. [77] and consensus sequences were subjected to further analyses. The consensus sequences for each DNA barcode region are shown in Table S3. The taxonomic assignment of *Stachys* sequences was performed through a BLAST search against publicly available accessions. The sequences for the DNA markers ITS, rbcL, and matK were retrieved from The Barcode of Life Data System (BOLD)’s database [78], which is enriched in conserved DNA fragments from different *Stachys* and a few *Betonica* species. Due to the lack of data about the trnH-psbA marker in BOLD, close sequences were pooled out from the NCBI database. The fragment length of all the sequences was adjusted to a region with a similar or close fragment length and the database sequences without differences in the analysed DNA barcoding region were discarded. The aligned sequence fragments were submitted for phylogenetic tree construction. Initial trees for the heuristic search were obtained by applying the maximum likelihood method and the Tamura–Nei model. The stability of the topology of the phylogenetic tree was assessed in the bootstrap test (500 replicates).

### 3.4. Genetic Stability Assay by SRAP Markers

To verify the genetic stability between in vitro and respective ex vitro adapted *S. scardica* plants, the sequence-related amplified polymorphism (SRAP) approach was applied. The procedure was performed according to Li and Quiros [35] and Zagorcheva et al. [36] and is described in detail by [11].

### 3.5. NMR Analyses

Samples of fresh plant material were air dried and 50 mg of each 5 replicates were homogenised with equal amounts of CD_3_OD (0.75 mL) and D_2_O (0.75 mL KH_2_PO_4_ buffer, pH 6.0) containing 0.005% (*w*/*v*) trimethylsilyl propanoic acid (TSPA-d4). After 30 min ultrasonication (35 kHz; UCI-50Raypa^®^ R. Espinar S.L., Barcelona, Spain), samples were centrifuged (13,000× *g*, 20 min); then, the supernatants were transferred to 5 mm glass-walled NMR tubes and analysed with the NMR spectrometer [79,80]. Briefly, proton (1H) as well as 2D NMR spectra (J-resolved, COSY, HSQC, and TOCSY) were recorded at 25 °C with an NEO600 spectrometer (Bruker, Karlsruhe, Germany) operating at a proton NMR frequency of 600.18 MHz [79]. Deuterated methanol was used for the internal lock. The resulting 1H NMR spectra for each sample were phased, baseline-corrected, and referenced to the residual signal of methanol-d4 at 3.30 ppm by running TopSpin software (4.1.4, Bruker BioSpin Group, Billerica, MA, USA). CD_3_OD and D_2_O used in the experiments were from Deutero GmbH (Kastellaun, Germany).

### 3.6. The Preparation of Methanolic Extracts

The crude methanolic extracts were obtained from 3 g of dried and finely powdered plant material from in situ wild, in vitro cultivated, and ex vitro adapted plants and subjected to triple ultrasound extraction with chloroform (Sigma-Aldrich, Madrid, Spain) for 10 min. Next, the biomass was extracted three times with methanol for 30 min. In the final step, the obtained crude extracts were concentrated with a vacuum evaporator (IKA, Königswinter, Germany) and dried to a constant dry weight. The yields of crude extracts from in situ, in vitro cultivated, and ex vitro adapted plants were 19.4%, 26.7%, and 19.8%, respectively.

### 3.7. The Determination of Total Phenolic and Flavonoid Contents

Total phenolic contents of *S. scardica* methanolic extracts were determined by the application of a Folin–Ciocalteu reagent according to the method described by Singleton et al. [81]. The content of total phenols was quantified using the curve of gallic acid as a standard and expressed as μg gallic acid equivalents per mg (μg GA/mg extract). The flavonoid determination was performed according to Chang et al. [82]. The flavonoid concentration was quantified by a standard curve using quercetin as the standard and expressed as μg quercetin equivalents per mg extract (μg QE/mg extract).

### 3.8. Antioxidant and Radical Scavenging Activity Assays

The total antioxidant activity (TAA) was determined by a modified method of Prieto et al. [83], described by Mantovska et al. [11]. The absorbance of the samples was measured at 695 nm on a spectrophotometer, Shimadzu 1800 UV. The TAA was expressed as mM α-tocopherol per gram extract (mM α-tocopherol/g extract).

The radical scavenging activity of methanolic extracts against the stable DPPH (1,1-diphenyl-2-picrylhydrazyl) and ABTS (2,20-azino-bis(3-ethylbenzothiazoline- 6-sulphonic acid) free radicals was determined by the methods described in detail by [11]. The results for DPPH scavenging potential were expressed as maximum % inhibition and 50% inhibition (IC_50_) concentration in μg/mL. The ABTS radical scavenging activity was presented as mg Trolox equivalents per mg extract (mg TE/mg extract).

The capacity of the extracts to reduce ferrous-containing radicals was determined according to the modified method of Benzie and Strain [84], described by Mantovska et al. [11]. Ferric-reducing antioxidant capacity was represented as mM Fe^2+^ using the FeSO_4_ standard curve.

### 3.9. Microtitre Hemolytic Complement Assay

The hemolytic complement assay was conducted using 96-well flat-bottom microtiter plates, following the protocol outlined by Mantovska et al. [11]. Initially, the dried methanolic extracts were dissolved in 3% dimethyl sulfoxide (DMSO) and then further diluted with barbitone-buffered saline (BBS) at pH 7.5 containing 0.15 mM of Ca^2+^. The assay involved a 6% suspension of sheep erythrocytes (SE) sensitised with rabbit polyclonal anti-SE serum and a guinea pig complement. To identify the dilution causing 50% haemolysis of the target erythrocytes, an initial titration of sera was performed. The SE (25 μL/well) was sensitised through a 30 min incubation with hemolysin (dilution 1:1600, 25 μL/well) at 37 °C. Subsequently, complements (125 μL/well of the appropriate dilution) and varying amounts of the analysed plant extracts (100 μL/well) were added, followed by a 1 h incubation at 37 °C. After centrifugation of the microtiter plates at 1000× *g* for 5 min at 4 °C, 200 μL of the supernatant from each well was transferred to new 96-well flat-bottom microtiter plates, and absorbance was measured at 540 nm using an ELISA reader (DR-200B, Hiwell Diatek Instruments, Wuxi, China). The results are presented as both maximum % inhibition and the concentration for 50% inhibition (IC50) in μg/mL. Each assay was performed in triplicate.

### 3.10. Antimicrobial Activity

The antimicrobial activity was assessed using the disc diffusion method, employing methanolic extracts from both in vitro and ex vitro cultivated *S. scardica* at concentrations of 8 mg/disk. Four bacterial test pathogens—*Bacillus cereus* ATCC 11778, *Escherichia coli* ATCC 25922, *Staphylococcus epidermidis* ATCC 12228, and *Propionibacterium acnes* (an isolate)—and pathogenic yeasts *Candida albicans* ATCC 18204 and *Malassezia furfur* ATCC 14521 were employed to evaluate the antimicrobial activity of the samples. Overlays of test pathogens (0.5 McFarland) were prepared on agar plates. Thirty microlitres of a pre-prepared solution of each sample was added to sterile disks to achieve a working concentration and allowed to diffuse. Control disks with 30 µL of 5% DMSO were used. The plates were incubated at the respective temperatures for each test pathogen, i.e., 37 °C and 30 °C for 24 h. Clear zones around the disks confirmed antimicrobial activity, and the diameters of the inhibition zones were measured in millimetres. The percentage of inhibition effect was calculated using the following formula: percentage of inhibition effect = (diameter of clear zone of the sample/diameter of clear zone of the positive control) × 100.

### 3.11. Data Analysis

The results were expressed as the mean ± standard error (SE) based on a minimum of 12 observations (3 repetitions per variant in each of 4 independent experiment sets). Statistical analysis was carried out using Sigma Plot 11.0 software, involving one-way ANOVA followed by the Holm–Sidak test with the significance level set at 0.001 to assess differences among all the variants.

## 4. Conclusions

The application of a biotechnological approach for inducing in vitro and ex vitro cultures of *S. scardica* allowed for the optimisation of protocols for the ex situ conservation of this endangered Balkan endemic species. Through established model systems, a comparative NMR metabolite profiling and exploration of the biological potential of methanolic extracts isolated from in situ wild, in vitro cultivated, and ex vitro adapted plants were achieved. Primary metabolites, such as organic acids and non-protein amino acids, likely participate in fine synchronisation with secondary metabolites in regulating plants’ adaptive response to changing environmental conditions. The metabolite profile of *S. scardica* revealed secondary metabolites typical for the genus, including phenylethanoid glycosides, phenolic acids, iridoids, and alkaloids. A trend of decreasing quantities of phenolic compounds and flavonoids, consequently affecting antioxidant, radical scavenging, antimicrobial, and anti-inflammatory potential, was observed during cultivation in aseptic conditions. However, the biosynthetic potential and associated biological activity were restored after adapting the plants to ex vitro conditions.

Our conducted research provides new insights into *Stachys* representatives and the biosynthesis of biologically active metabolites under changing environmental conditions. Subsequent studies focusing on unveiling the mechanism of action of isolated extracts and/or fractions in animal in vitro and in vivo model systems could contribute to a comprehensive characterisation of the biological potential of this species. Additionally, it could establish an innovative and sustainable platform for the biosynthesis of pharmaceutically significant metabolites through the selection of in vitro cultured/ex vitro adapted lines with high biosynthetic potential.

## Figures and Tables

**Table 1:** Effect of different concentrations of benzyladenine BA (0.1–2.5 mg/L) on biomass, shoot and root formation, and callusogenesis of in vitro propagated *S. scardica* plants.

Variants	Fresh Weight [g]	Dry Weight [g]	Number of Shoots	Number of Internodes	Growth Index	Root Length [cm]	Callus Formation
**Control**	0.34 ± 0.2	0.05 ± 0.0	2.2 ± 0.40	1.54 ± 0.47	3.3 ± 1.1	5.73 ± 1.6	-
**0.1 mg/L BA**	0.18 ± 0.04	0.04 ± 0.02	2 ± 0.00	1.5 ± 0.5	3.0 ± 1.0	-	-
**0.5 mg/L BA**	0.53 ± 0.10	0.075 * ± 0.01	3.2 * ± 0.4	1.56 ± 0.25	5.0 ± 1.0	-	-
**1 mg/L BA**	0.76 * ± 0.28	0.085 ± 0.02	6.4 * ± 1.2	1.67 ± 0.16	10.8 ± 2.8	-	+ ^1^
**1.5 mg/L BA**	0.98 * ± 0.16	0.083 * ± 0.01	16.2 * ± 2.0	1.3 * ± 0.25	22.6 ± 2.4	-	+ ^1^
**2 mg/L BA**	0.9 * ± 0.07	0.08 * ± 0.01	17.3 * ± 3.1	1.5 ± 0.29	24.0 ± 8.6	-	+ ^2^
**2.5 mg/L BA**	1.38 * ± 0.22	0.12 * ± 0.01	15.2 * ± 2.7	1.6 * ± 0.35	23.8 ± 3.2	-	+ ^2^

Mean values (±SD) and significant changes are indicated with asterisk * (*p* ≤ 0.05). ^1^ Slight callus formation; ^2^ significant callus formation.

**Table 2:** Best identity scores (%) of *S. scardica* against database accessions.

	rbcL	matK	trnH-psbA	ITS
***S. scardica***	*S. officinalis*	*S. officinalis*	*B. officinalis*	*B. officinalis*
	(100%)	(100%)	(99%)	(84%)
	*S. macrantha*	*S. macrantha*		*B. macrantha*
	(99%)	(99%)		(87%)
			*B. alopecuros*	*S. alopecuros*
			(96%)	(86%)

**Table 3:** Metabolites identified in *S. scardica* plants by relevant 1D and 2D NMR spectra.

Metabolite	*S. scardica* In Situ	*S. scardica* In Vitro	*S. scardica* Ex Vitro	Selected Signals, Multiplicity and Coupling Constant ^a^
Alanine	+	+	+	*δ* 1.47 (d, *J* = 7.2)
Glutamine		+		*δ* 2.13 (m)/*δ* 2.45 (m)/*δ* 3.74 (t)
α-Glucose	+	+	+	*δ* 5.17 (d, *J =* 3.8)
β-Glucose	+	+	+	*δ* 4.56 (d, *J =* 7.9)/3.18 (dd, *J =* 7.9, 9.2)
Sucrose	+	+	+	*δ* 5.37 (d, *J =* 3.8)
Acetic acid	+		+	*δ* 1.92 (s)
Lactic acid		+		*δ* 1.31 (d, *J* = 6.9)/*δ* 4.08 m
Succinic acid	+		+	*δ* 2.48 (s)
Formic acid	+	+	+	*δ* 8.45 (s)
Malic acid		+		*δ* 2.80 (dd, *J* = 16.9, 8.2)/*δ* 2.93 (dd, J= 16.9, 3.9)
Choline		+		*δ* 3.19 (s)
Allobetonicoside		+		*δ* 6.46 (d, *J* = 6.4)/*δ* 6.10 (bs)/*δ* 5.92 (d, *J* = 1.3)/ *δ* 5.30 (d, *J* = 8.3)/*δ* 5.02 (dd, *J* = 6.4, 1.2)/*δ* 4.71 (d, *J* = 7.8)/*δ* 2.33 (s)
8-OAc-harpagide		+		*δ* 6.42 (d, *J* = 6.5)/*δ* 6.04 (d, *J* = 1.3)/*δ* 4.96 (dd, *J* = 6.5, 1.7)/ *δ* 4.67 (d, *J* = 7.9)/*δ* 2.04 (s)/*δ* 1.43 (s)
Verbascoside	+	+	+	*δ* 7.63 (d, *J* = 15.9)/*δ* 7.14 (d, *J* = 2.0)/7.05 (dd, *J* = 8.3, 2.0)/ *δ* 6.67 (dd, *J =* 8.3, 2.0)/*δ* 6.34 (d, *J* = 15.9)/4.93 (t, *J* = 9.6)/4.47 (d, *J* = 7.9)/ *δ* 2.81 (t, *J* = 7.2) 1.04 (d, *J* = 6.4)
Leucosepthoside A	+	-	+	*δ* 7.70 (d, *J* = 15.8)/*δ* 7.23 (d, *J* = 1.9)/7.16 (dd, *J* = 8.3, 2.0)/ *δ* 6.89 (dd, *J =* 8.3, 2.0)/*δ* 6.41 (d, *J* = 16.0)/4.93 (t, *J* = 9.6)/4.47 (d, *J* = 7.9)/ *δ* 3.88 (s)/*δ* 2.81 (t, *J* = 7.1) 1.04 (d, *J* = 6.4)
Chlorogenic acid	+	+	+	*δ* 7.60 (d, *J* = 15.7)/*δ* 7.13 (d, *J* = 2.2)/*δ* 7.06 (dd, *J* = 8.2, 2.2)/ *δ* 6.86 (d, *J* = 8.3)/*δ* 6.33 (d, *J* = 15.9)/*δ* 5.30 (td, *J* = 4.9, 10.9)/*δ* 4.18 (br q, *J* = 3.1)
Trigonelline	+	-	+	*δ* 9.12 (s)/*δ* 8.83 (m)/*δ* 8.07 (m)/*δ* 4.43 (s)

The sign “+” refers to relative fold differences and “-” refers to absence of the particular compound. ^a^ Proton NMR chemical shifts (*δ* in ppm) in CD_3_OD/D_2_O and coupling constant (*J* in Hz); reference CD_3_OD (*δ* 3.30 ppm).

**Table 4:** Antibacterial activity of methanolic extracts (8 mg per disk) from in vitro cultivated and ex vitro adapted *S. scardica* plants.

Test Strain	Inhibiton Zone (mm)				
	(+) Control		5% DMSO	*Stachys scardica* In Vitro	*Stachys scardica* Ex Vitro
***Bacillus cereus*** **ATCC 11778**	Gentamicin	19.17 ± 0.45	NZ	8.09 ± 0.11 ^c^	12.43 ± 0.04 ^c^
	10 µg/disk				
***Staphylococcus epidermidis*** **ATCC 12228**	Gentamicin	24.51 ± 0.42	NZ	8.74 ± 0.21 ^c^	24.93 ± 0.78 ^a^
	10 µg/disk				
***Propionibacterium acnes*** **(an isolate)**	Clindamycin	17.65 ± 0.50	NZ	7.78 ± 0.16 ^c^	26.58 ± 0.55 ^c^
	2 µg/disk;				
***Escherichia coli*** **ATCC 25922**	Gentamicin	18.74 ± 0.59	NZ	NZ	NZ
	10 µg/disk				
***Malassezia furfur*** **ATCC14521**	Nystatin	17.38 ± 0.77	NZ	NZ	NZ
	100 units/disk				
***Candida albicans*** **ATCC10231**	Nystatin	22.0 ± 0.09	NZ	NZ	NZ
	100 units/disk				

NZ—no inhibition zone. Values are presented as mean ± standard deviation. Statistical analysis was conducted using ANOVA, followed by the post hoc Tukey test: ^a^—nonsignificant (*p* > 0.05); ^c^—significant *p* < 0.01.

## Data Availability

Data are contained within the article and supplementary materials.

## Supplementary Materials

The following supporting information can be downloaded at https://www.mdpi.com/article/10.3390/plants13010030/s1: Table S1: Summary of SRAP alleles data following SRAP analysis of *S. scardica*; Table S2: PCR primers for DNA barcoding markers; Table S3: Sequences information for DNA barcoding of *S. scardica*. References [85,86,87,88,89,90] are cited in the supplementary materials.
