# Supplementary material for: Exploring the Phytochemical Composition and Biological Potential of Balkan Endemic Species Stachys scardica Griseb
Source: Plants (Basel). 2023 Dec 21;13(1):30. doi: 10.3390/plants13010030 (PMC10780532; doi:10.3390/plants13010030)
Supplement: Supplementary file 1 [file plants-13-00030-s001.zip › plants-2779963-supplementary.pdf]

Supplementary Materials

Table S1. Summary of SRAP alleles data following SRAP analysis of *S. scardica*.

**Primer Pair**	**SRAP Alleles for *S. scardica***
ME1_EM3	59
ME1_EM5	30
ME1_EM6	25
ME1_EM7	40
ME1_EM10	38
ME3_EM2	27
ME3_EM5	47
ME4_EM5	33
ME6_EM3	49
ME6_EM5	40
ME7_EM2	24
ME8_EM1	20
ME8_EM8	71
ME10_EM4	31
ME10_EM7	34
ME10_EM9	19
Total	587

Table S2. PCR primers for DNA barcoding markers.

DNA Barcoding Marker	Primers	Sequences	PCR Program	Ref.
ITS 1	ITS5AF<br>ITS4R	CCTTATCATTTAGAGGAAGGAG<br>TCCTCCGCTTATTGATATGC	94°C 5 min<br>[94°C 30 s,<br>50°C 30 s,<br>72°C 1min]<br>- 35 cycles<br>72°C 5 min	[85]
rbcL	rbcLa-F<br>rbcLajf634R	ATGTCACCACAAACAGAGACTAAAGC<br>GAAACGGTCTCTCCAACGCAT	94°C 4 min<br>[94°C 30 s<br>55°C 30 s<br>72°C 1 min]<br>- 35 cycles<br>72°C 10 min	[86]<br>[87]
matK	matK-KIM1R<br>matK-KIM3F	ACCCAGTCCATCTGGAAATCTTGGTTC<br>CGTACAGTACTTTTGTGTTTACGAG	95°C 5 min<br>[95°C 30 s,<br>51°C 50 s<br>72°C 1 min]<br>- 35 cycles<br>72°C 7 min	[88]
trnH-psbA	trnH-F<br>psbA3_r	CGCGCATGGTGGATTCACAATCC<br>GTTATGCATGAACGTAATGCTC	94°C 4 min<br>[94°C 30 s,<br>55°C 30 s,<br>72°C 1 min]<br>- 35 cycles<br>72°C 7 min	[89]<br>[90]

Table S3. Sequences information for DNA barcoding of *S. scardica.*

DNA Barcoding Marker	Sequence 5′-3′ of *Stachys* sp., Sequence Length (bp)	BOLDSystems ID
ITS,<br>*694 bp*	AACCTGCGGAAGGATCACTGTCGAAACCTGCAAAGAAGATCACGAACACGTTTCGAACA<br>ACCCCAGGCGCCGCGGTGCGGGGGGAGACCCCCGTCGAGGCCCCGAACCCATCGACGCGC<br>TCGCTAGCATTCACACCGCGCGGRCTAATGAACTCGGGCGCGGGATGCGCCAAGGAAAAT<br>GAAACGTAGCACACCCCTACCCGCCTCGCCTCATCAGCAGGGCGAAGGGAAGCAGGGGGC<br>ACCTATCGAATGTCTAAATGACTCTCGACAACGGATATCTCGGCTCCCGCATCAATGAAG<br>AACGTAGGGAAATGTTATACTTGGTGTGAATTGTAGAATCCCGTGAACCATCGAGTCTTT<br>GAACGCAAGTTGCGCCCAAAGCCATTAGGCCGAGGGCACGTCTGCCTGGGCGTCACGTAT<br>CACGTTGCCCCCCTCCCCCGCAGGGGCTGGGGGAAGAGATTGGCCTCCCGTGCGCCTCCA<br>GGCTGCGCGGTCGACCCAAATGCGAATCCGCCGTCGATGCACATCGCGACCAGCGGTGGT<br>TAAACTATTAACTCTCGTGCTGTCGTGTACCAAGGCATCCTCGRTCCGAAAATGTCCACG<br>AAGGACCCAATGGCGCGCAAGCACCCACGGCCGCGACCCCAGGTCAGGCGGGATCACCTG<br>CTGAGTTTAAGCATATCAATAAGCGAAGTAAAAAA	BUL003-23.ITS
rbcL,<br>*574 bp*	AGAGACTAAAGCAAGTGTTGGATTCAAAGCGGGTGTTAAAGAGTACAAATTGACTTATTA<br>TACCCCTGAATACGAAACCAAAGATACTGATATCTTGGCAGCATTCCGAGTAACTCCTCA<br>ACCTGGAGTTCCGCCCGAAGAAGCAGGGGCCGCGGTAGCTGCCGAATCTTCGACTGGTAC<br>ATGGACAACTGTGTGGACCGATGGACTTACCAGTCTTGATCGTTACAAAGGGCGATGCTA<br>CCACATTGAGCCCGTTCTTGGAGAAAAAGATCAATATATCTGTTATGTAGCTTACCCTTT<br>AGACCTTTTTGAAGAAGGTTCTGTTACTAACATGTTTACTTCCATTGTAGGAAATGTATT<br>TGGATTCAAAGCCCTACGTGCTCTACGTCTGGAAGATCTGCGAATCCCTACTGCTTATAC<br>TAAAACTTTCCAAGGCCCGCCCCATGGGATCCAAGTTGAGAGAGATAAATTGAACAAGTA<br>TGGTCGTCCTCTGTTGGGATGTACTATTAAACCGAAATTGGGGTTATCTGCTAAAAACTA<br>TGGTAGAGCGGTTTATGAATGTCTTCGCGGTGGA	BUL003-23.rbcL
matK,<br>*859 bp*	TCTTGGTTCAAATCCTTCGCTATTGGGTAAAAGATGCTTCCTCCTTGCATTTATTACGAG<br>TCTTTCTCAACGAATATTGTAATTGGAATAGTCTTTTTATTCCAAAGAAAGCCAGTTCCC<br>CTCTTTCAAAAAAAAATAAACGATTATTCTTATTCTTATATAATTCTCATGTATGTGAAT<br>ATGAATCCATTTTCGTCTTTCTACGTAACCAATCTTTTCATTTACGATCAACATCTTCTG<br>GAGTTTTTCTTGAACGAATCTATTTCTATATAAAAATAGAACGTCTTGTGAACGTCTTTG<br>TTAAAATTAAGGATTTTCGGGCACACCCGCGGTTGGTCAAGGAACCTTTCATGCATTATA<br>TTAGGTATCAAAAAAGATCCATTCTGGCTTCAAAAGGGACATTTCTTTTCATGAAGAAAT<br>GGAAATTGTACCTTGTCACTTTTTGGCAATGGCATTTTTCGTTGTGGTTTCATCCAAGAA<br>GGATTTATATAAACCAATTATCCAAGCATTCCCTTGCGTTTTTGGGCTATCTTTCAAGCG<br>TGCGAATGAATCCTTCCGTAGTACGCAGTCAAATTCTAGAAAATTCATTTCTGATCAATA<br>ATGCTATTAAAAAGTTTGAGACTCTTGTTCCAATTATTCCTCTGATTGCGTCATTGGCTA<br>AAGCCAAATTTTGTAACGTATTGGGGTATCCCGTTAGTAAGCCGATTCGGGCTGATTTAT<br>CAGATTCTAATATTATTGACCGGTTTGGGCGCATATGCAGAAATCTTTCTCGTTATCATA<br>GTGGATCTTCCAAAAAAAAGAGTTTGTATCGAATAAAGTATATACTTCGACTTTCTTGCG<br>CTAGAACTTTGGCTCGTAA	BUL003-23.matK
trnH,<br>*472 bp*	ATTCACAATCCACTGCCTTGATCCACTTGGCTACATCCGCCCCTCTACTATTACTTAGTA<br>TTAATGAATAATGAATTTAGTTTGAAATTCAATTTCAATCATTTTGATTTCGTCTTTCTT<br>TTTTATCTTTAAAAATCAAAAACTTACCAACCCTTTTTACAAGAAGTTAATTAAATGAAA<br>CTAAAAAAAATTATACAGAACAAAAAAAGAATATTCAATCATTAATCAATCCTTAAGAAT<br>CCCCCTCTTTCTTTTTCTAGATCCTGTAATGGAAAGAATGGAAAGAAAAAACGTAATTGA<br>AAAGCAAATTAAGGAGCAATAAACTCTTTCTTGTTTTATCAAGAGGGGTTATTGCTCCTT<br>AATTTTATCTATTCTATTTTCAAAAACGCCTATACACTAAGACCAAGTCTTATCCATTTG<br>TTGGAGCTTCGATAGAAGCTAGGTCTAGAGGGAAGTTATGAGCATTACGTTA	BUL003-23.trnH-psbA
